# A Study of Hydrogen Embrittlement of SA-372 J Class High Pressure Hydrogen Storage Seamless Cylinder (≥100 MPA)

**DOI:** 10.3390/ma15217714

**Published:** 2022-11-02

**Authors:** Ruifeng Yin, Ruidong Fu, Ningning Gu, Yongjiu Liu

**Affiliations:** 1 State Key Laboratory of Metastable Materials Science and Technology, Yanshan University, Qinhuangdao 066004, China; 2 College of Materials Science and Engineering, Yanshan University, Qinhuangdao 066004, China; 3 Shijiazhuang Enric Gas Equipment Co., Ltd., No. 169 Yuxiang Street, Shijiazhuang 051430, China

**Keywords:** SA-372 grade J steel, hydrogen storage cylinder, hydrogen embrittlement index, disc test, hydrogen embrittlement

## Abstract

The spinning process will lead to changes in the micro-structure and mechanical properties of the materials in different positions of the high-pressure hydrogen storage cylinder, which will show different hydrogen embrittlement resistance in the high-pressure hydrogen environment. In order to fully study the safety of hydrogen storage in large-volume seamless steel cylinders, this chapter associates the influence of the forming process with the deterioration of a high-pressure hydrogen cylinder (≥100 MPa). The anti-hydrogen embrittlement of SA-372 grade J steel at different locations of the formed cylinders was studied experimentally in three cylinders. The hydrogen embrittlement experiments were carried out according to method A of ISO 11114-4:2005. The relationship between tensile strength, microstructure, and hydrogen embrittlement is analyzed, which provides comprehensive and reliable data for the safety of hydrogen storage and transmission.

## 1. Introduction

Hydrogen energy is recognized as one of the most effective ways to solve the global energy crisis, climate deterioration, and environmental pollution. In the whole industry chain of hydrogen energy, hydrogen storage and transportation are key links to restricting the development of hydrogen energy and the fuel cell industry in China [1,2,3]. The hydrogenation station is the core infrastructure to provide hydrogen for hydrogen fuel cell vehicles and other hydrogen energy-utilizing devices. The development of hydrogen energy puts forward harsh requirements for safe and efficient hydrogen storage and transportation systems [4,5,6,7]. In order to achieve fast charging, the ideal hydrogen storage pressure of the hydrogenation station should be above 100 MPa. With the increase in hydrogen pressure, the problem of hydrogen embrittlement becomes more prominent. The hydrogen embrittlement of metal materials at room temperature and high pressure must be considered to ensure their long-term, stable, and reliable operation. Hydrogen embrittlement is the phenomenon of material plasticity reduction and embrittlement caused by hydrogen.

Hydrogen embrittlement in Cr-Mo steel under normal temperature and high pressure has been a topic of considerable interest and many research efforts have focused on the topic. In the evaluation of material hydrogen embrittlement, there is a mature international standard or method [8,9,10,11,12,13,14,15,16]. The publicly available data to support these fatigue- based designs were summarized in just a handful of publications, in particular research by [17,18]. Wada et al. [19] point out that for CSM440 at 45 MPa, ultra-high purity hydrogen at room temperature, the hydrogen brittleness resistance test was carried out. Tensile test results show that the yield strength and maximum tensile strength are not different from those in air, but the toughness is decreased. The measurement of fatigue crack growth rates in gaseous hydrogen at the different design pressures were performed. The fatigue used in the development of the master curve for high-pressure gaseous hydrogen service were reported in several studies in Cr-Mo [20,21] and Ni-Cr-Mo [22,23]. However, there is no research on the mechanism of different hydrogen embrittlement caused by different spinning processes in a high pressure (≥100 MPa) hydrogen environment.

In this paper, the forming process of large volume seamless hydrogen storage cylinders is usually a spinning process, followed by quenching and tempering to improve the mechanical properties. The spinning process results in changes in the structure and mechanical properties of materials at different locations in the cylinder, which results in different anti-hydrogen brittleness properties under a high-pressure hydrogen environment. To fully study the safety of hydrogen storage in large-volume seamless steel cylinders, this chapter associates the influence of the forming process with the deterioration of high-pressure hydrogen (≥100 MPa). The anti-hydrogen embrittlement of SA-372 grade J steel at different locations of the formed cylinders is studied experimentally for three cylinders. The hydrogen embrittlement experiments were carried out according to method A of ISO 11114-4:2005.

## 2. Test Method and Sample Preparation

### 2.1. Summary of the Test Method

The disc test is the blasting of disc samples at a constant explosion rate. The resistance of material to hydrogen embrittlement is evaluated by comparing the ratio between hydrogen blasting pressure P_He_ and helium blasting pressure P_H2_, in which helium is used as a reference gas.

In evaluating the embrittlement index of a material, ISO 11114-4:2005 introduced that if the maximum value of the above-mentioned ratio is less than or equal to 2, it should be considered suitable for high pressure hydrogen gas cylinders.

I = P_He_/P_H2_ < 2 (1)

### 2.2. High-Pressure Hydrogen Storage Cylinder Manufacturing

The primary focus of this work is high pressure hydrogen storage cylinder steel SA-372 Grade J, in particular quenched and tempered (Q&T) high pressure hydrogen storage cylinders for hydrogen service at a pressure above 100 MPa.

**Figure 1:**
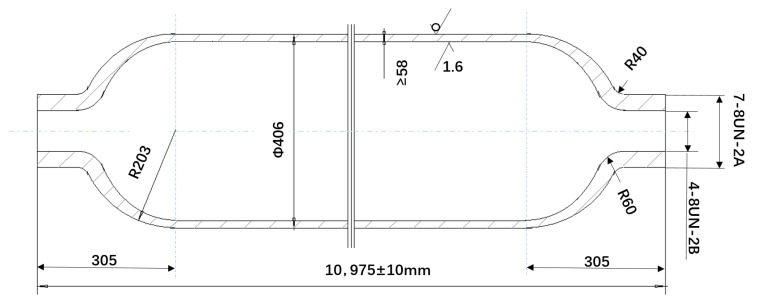
The dimensions of the high-pressure hydrogen storage cylinder.

The disc test sample was taken from a 103 MPa high-pressure hydrogen storage cylinder produced by a company in China. Its structure is shown in Figure 1.

**Figure 2:**
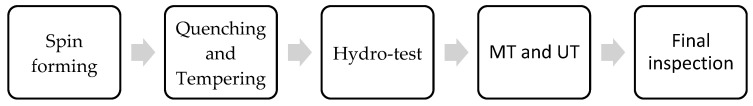
Manufacturing process.

The main manufacturing process of the 103 MPa high-pressure hydrogen storage cylinder is shown in Figure 2. These experimental heat treatment materials have the same composition as below in Table 1.

### 2.3. Sampling Location and Numbers

#### 2.3.1. Disc Sampling (A, B, and C)

**Figure 3:**
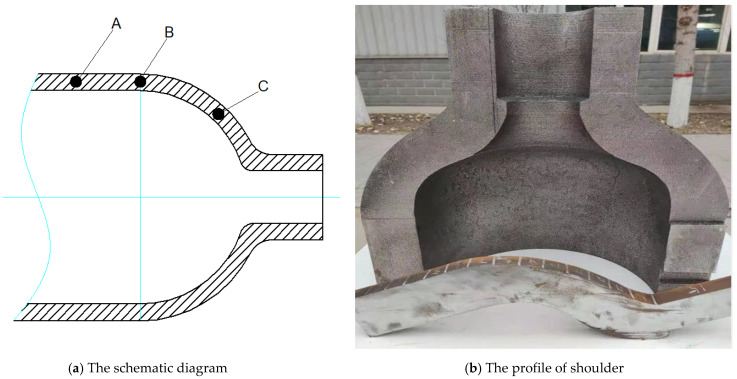
Sampling locations. The blue lines is center line.

Disc sampling (A, B, and C) is shown in Figure 3 after spinning the cylinder. The sampling direction is circular. The sampling positions are the cylinder body, shoulder and the joint of the cylinder body. The sampling depth is 1/2 wall thickness from the surface.

#### 2.3.2. Sampling Disc

The sample disc should be flat and ground (or machined to an equivalent surface finish) and have the following characteristics and dimensions:

- Diameter: 58 ± 0.05 mm;
- Thickness: 0.75 mm ± 0.005 mm;
- Flatness: less than 1/10 mm deflection.

Surface condition (both sides): The value of Ra is less than 0.001 mm. The disc test samples used for H_2_ and He measurements should have the same roughness. No trace of oxide. To verify the sample quality, immediately store the sample in a dry environment, such as a dryer. After final preparation and before testing, degrease the samples and check the thickness at four points 90 degrees apart to determine the average thickness. Determine whether the disc’s hardness altered the original material properties.

#### 2.3.3. Number of Samples

The tests must be performed with hydrogen (>99.9995%) and helium (H20 < 3 μL/L) for a range of pressure rise rates evenly distributed between 0.1 and 1000 bar/min. When these lower pressure rates are used, the minimum breaking pressure should be determined. Minimum values for running the remaining tests should be used. The sample is taken out of three cylinders, each with three tensile samples. It is generally considered that 6 helium tests and 9 hydrogen tests (15 tests in total) per cylinder are sufficient for a thorough material evaluation.

See Table 2 for the number of disc and tensile test samples at the cylinder, shoulder, and the junction of cylinder and shoulder.

### 2.4. Test Method

**Figure 4:**
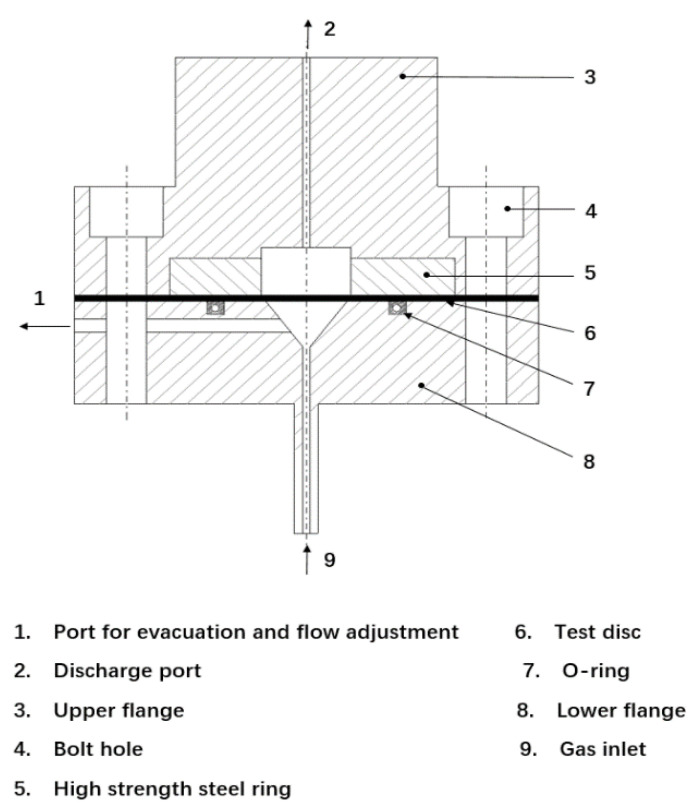
Test apparatus.

The schematic diagram of the disc pressure test method apparatus for hydrogen brittleness testing of high-pressure cylinder steel is shown in Figure 4.

The upper and lower stainless steel flanges are connected by high-strength bolts with a tensile strength of 1100 MPa. The volume of the lower chamber is about 5 cm^3^, and the maximum inner diameter of the conical surface is 25.5 mm. The diameter of the superior cavity is 25.5 mm. The fillet diameter of the high-strength steel ring is 0.5 mm. In the test facility, the “vent and flow control outlet” and “discharge outlet” ensure the safe discharge of the test gas through pipeline connection.

The hydrogen embrittlement sensitivity test is mainly tested by the disk test. By measuring the disk bursting pressure in the environment of hydrogen and inert gas, the relative bursting pressure value is obtained, and the hydrogen embrittlement of the material is determined according to the value.

The disc-shaped specimens are subjected to increasing gas pressure at a constant rate and thus rupture. With helium as reference gas, the embrittlement effect of hydrogen is proved by comparing the hydrogen breaking pressure P_H2_ with the helium breaking pressure P_He_. The ratio of P_He_/P_H2_ should be determined. The lower the ratio, the better the steel section will perform in the presence of hydrogen. This ratio depends on the rate of pressure rise and should remain constant throughout the test.

## 3. Test Results and Discussion

### 3.1. Helium Rupture

All the testing considered in this overview was conducted at room temperature (20 °C). The helium rupture test results are shown in Table 3, where the corrected helium rupture pressure is calculated as below:

(2) $$P_{r}^{\prime }=\frac{P_{r}\times 0.75}{e_{m}}$$

where, e_m_ is the average disc thickness, Pr is the rupture pressure, and Pr′ is the corrected rupture pressure.

**Figure 5:**
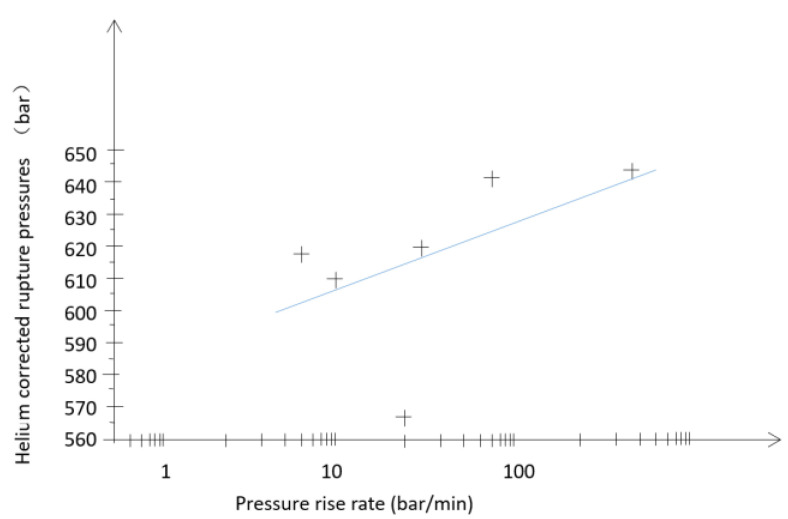
Variation in corrected helium rupture pressures as a function of the p ressure rise rate. The + sign indicate helium rupture pressure in different pressure rise rate.

Figure 5 depicts the corrected helium rupture pressure as a function of the pressure rise rate.

The regression curve can be expressed by the following equation:

He = 584.20 + 22.16log(x)(3)

### 3.2. H2 Rupture Pressure Test

#### 3.2.1. Three Cylinder Body P_r_′_He_/P_r_′_H2_ Ratio

The corrected rupture pressure is compared with the average rate of pressure rise (actual rupture pressure divided by test duration; expressed in bar/min) and for each hydrogen test of three cylinder bodies. The variation in the ratio of Pr′_He_/Pr′_H2_ as a function of the pressure rise rate is shown in Table 4.

Pr′_He_ is the theoretical helium rupture pressure corresponding to the same pressure rise rate as that for the hydrogen test, which is calculated from the regression equation of the corrected helium rupture pressure. Pr′H2 is the corrected hydrogen rupture pressure.

#### 3.2.2. The P_r_′_He_/P_r_′_H2_ Ratio of Three Cylinders at the Body-Shoulder Junction

The corrected rupture pressures are plotted against the mean pressure rise rate (actual rupture pressure divided by the test duration; expressed in bar/min), and for each hydrogen test of the junction of body and shoulder. The variation in the ratio of Pr′He/Pr′H2 as a function of the pressure rise rate is shown in Table 5.

Pr′_He_ is the theoretical helium rupture pressure, which corresponds to the same boost rate as the hydrogen test and is calculated from the modified helium rupture pressure regression equation. Pr′_H2_ is the corrected hydrogen rupture pressure.

#### 3.2.3. The P_r_′_He_/P_r_′_H2_ Ratio of the Shoulder of the Three Cylinders

The corrected rupture pressures are plotted against the average pressure rise rate (actual rupture pressure divided by the test duration; expressed in bar/min), and for each hydrogen test of the shoulder of the three cylinders. The variation in the ratio of Pr′_He_/Pr′_H2_ as a function of the pressure rise rate is shown in Table 6.

**Figure 6:**
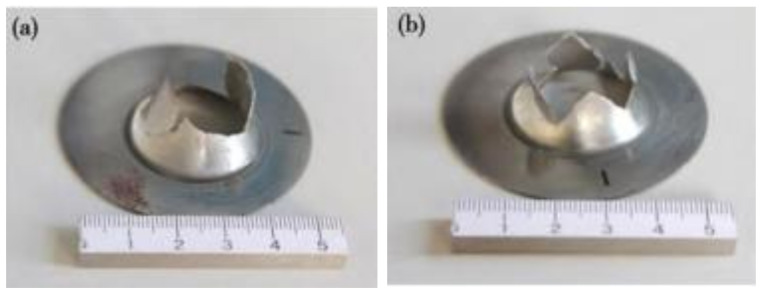
Blasted specimen after disk test (**a**) He environment and (**b**) H2 environment.

In the comparison of hydrogen embrittlement sensitivity coefficients, the minimum P_He_/P_H2_ is 1.38 at a pressurization rate of 62.2 MPa/min and the maximum is 1.88 at a pressurization rate of 6.0 MPa/min. Figure 6 shows the disc specimen blasted in helium and hydrogen environments.

### 3.3. Analysis of Experimental Results

By comprehensively comparing Table 4, Table 5 and Table 6, the hydrogen embrittlement index of SA-372 grade J high-pressure hydrogen cylinders (100 MPa) is as follows:

Cylinder body > the junction of body and shoulder > cylinder shoulder.

#### 3.3.1. The Relationship between the Strength and the Sensitivity to Hydrogen Embrittlement Index

From Table 7, it can be analyzed that the higher the SA-372 Grade J strength level of the high-pressure hydrogen storage bottle, the higher the hydrogen embrittlement index and the worse the hydrogen embrittlement.

**Figure 7:**
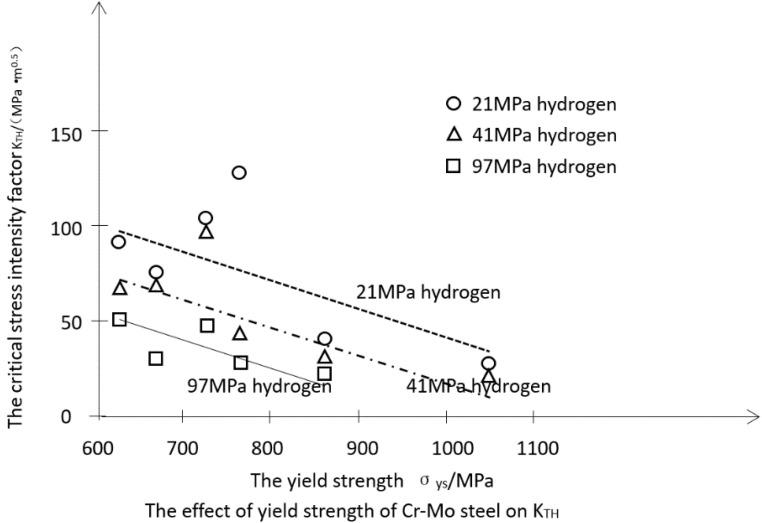
Effect of yield strength on the K_TH_ of chrome-molybdenum steel. The “Solid line” refers to the 97 MPA high-pressure hydrogen environment, the “dashed line” indicates in the 41 MPA high-pressure hydrogen environment, the “dotted line” indicates in the 21 MPA high-pressure hydrogen environment. K_TH_ is the critical stress intensity factor in the fracture mechanics evaluation.

The results are basically equivalent to the study by Nelson et al. [24], who showed that in the low-pressure hydrogen environment (0.08 MPa), the yield strength of 4130 steel increases from 1050 to 1330 MPa. K_TH_ decreases from 60 to 20 MPa·m^0.5^ left to right. At a temperature of 13 ℃ and in high-pressure hydrogen gas environments (21 MPa, 41 MPa, and 97 MPa), the critical stress intensity factor K_TH_ of chromium-molybdenum steel (AISI 4130, 4145, and 4147) also shows a similar trend [25,26,27]. As shown in Figure 7, the higher strength steels exhibit higher elastic strain when plastic deformation occurs, resulting in lower stress intensity required for crack growth with higher notch sensitivity at the bottom of the defect or crack, which results in higher hydrogen brittle sensitivity, i.e., the higher the strength level, the lower the critical stress intensity factor K_TH_. AISI 4130, 4145, and 4147 are chromium-molybdenum steels commonly used in high-pressure hydrogen systems.

Hinotani et al. [28] showed that in the high purity hydrogen environment with a test pressure of 19.6 MPa, when the tensile strength of high manganese steel is reduced to below 882 MPa and the tensile strength of chromium-molybdenum steel (AISI4130) and chromium-nickel-molybdenum steel (AISI4340) is reduced to below 950 MPa, the K_TH_ value increases significantly and the hydrogen brittleness sensitivity decreases significantly.

#### 3.3.2. The Relationship between Different Microstructures and the Hydrogen Embrittlement Index

**Figure 8:**
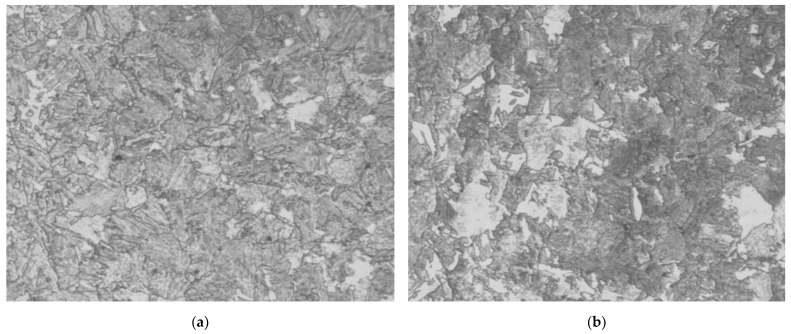
Microstructure of a cylinder (**a**) before spinning and (**b**) after spinning.

The microstructure of the cylinder body and spinning shoulder of the hydrogen storage cylinder is shown in Figure 8. After quenching and tempering, the microstructure of the cylinder body of the hydrogen storage cylinder transforms into tempered sorbite and a little bainite, and the microstructure of the end of the hydrogen storage cylinder transforms into tempered sorbite and balanced sorbite.

From Table 7, it can be analyzed that the higher the SA-372 Grade J strength level of the high-pressure hydrogen storage cylinder compared with tempered sorbite and balanced sorbite, the tempered sorbite and a little bainite have a lower hydrogen embrittlement index and better hydrogen embrittlement resistance.

The results are basically equivalent to the study of Yang Zhikang [29] who tested different structures of eight kinds of carbon steel and alloy steel, and came to the conclusion that the sensitivity of different structures to hydrogen brittleness is ranked from large to small as follows: original martensite > low temperature tempered martensite > tempered troostite with original martensite posture orientation > bainite > tempered sorbite (high temperature tempering) > balanced sorbite (isothermal quenching) > pearlite (high temperature annealing).

**Figure 9:**
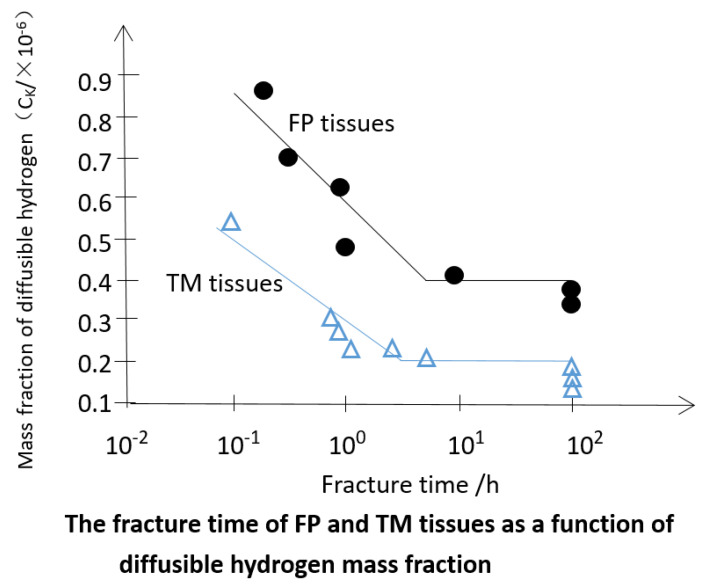
The fracture time of FP tissue (Black dot) and TM tissues (blue triangle).

The variation of the delay fracture time of notched samples with two kinds of tissues as a function of the mass fraction of diffusible hydrogen in steel is shown in Figure 9.

It can be seen from Figure 9 that the critical mass fraction of hydrogen C_K_ in TM and FP tissues that initiates crack propagation is 0.2 × 10^−6^ and 0.41 × 10^−6^, respectively. The authors of [30] studied ultra-high strength steels and showed that at the same tensile strength (1600 MPa), the martensite (TM) tempered at 450 ℃ had a higher sensitivity to hydrogen-induced delayed crack than the all-perlite structure (FP) [30,31]. Under the same diffusible hydrogen mass fraction, the maximum fracture stress of FP tissue is higher than that of TM tissue. In addition, the equilibrium saturation concentration of diffusible hydrogen in FP tissue is higher than that in TM tissue.

## 4. Conclusions

SA-372 grade J steel in different locations of the spinning cylinders (≥100 MPa) was studied in the hydrogen embrittlement experiments. They were carried out according to method A of ISO 11114-4:2005.

1. The experimental results show the hydrogen embrittlement index is cylinder body > the junction of body and shoulder > cylinder shoulder > 2;
2. The experimental results show an increase in the strength of SA372 Grade J steel will lead to an increase in hydrogen embrittlement sensitivity;
3. The experimental results show that different microstructures have different hydrogen embrittlement sensitivity. The hydrogen embrittlement sensitivity of balance sorbite is better than that of pearlite.

## Figures and Tables

**Table 1:** The chemical composition of SA-372 Grade J.

Chemical Element	C	Mn	P	S	Si	Cr	Mo
Mass fraction %	0.39	0.90	0.010	0.002	0.31	1.07	0.22

**Table 2:** The total number of disc and tensile test samples.

Sampling Location	Disc Test Sample No.	Tensile Test Sample No.
Cylinder body	Cylinder 1: A1_1_, A1_2_, A3_1_–A3_3_ Cylinder 2: A2_1_, A2_2_, A3_4_–A3_6_ Cylinder 3: A3_1_, A3_2_, A3_7_–A3_9_	Cylinder 1: D1–D3 Cylinder 2: D4–D6 Cylinder 3: D7–D9
Shoulder	Cylinder 1: B1_1_, B1_2_, B3_1_–B3_3_ Cylinder 2: B2_1_, B2_2_, B3_4_–B3_6_ Cylinder 3: B3_1_, B3_2_, B3_7_–B3_9_	Cylinder 1: E1–E3 Cylinder 2: E4–E6 Cylinder 3: E7–E9
The junction of body and shoulder	Cylinder 1: C1_1_, C1_2_, C3_1_–C3_3_ Cylinder 2: C2_1_, C2_2_, C3_4_–C3_6_ Cylinder 3: C3_1_, C3_2_, C3_7_–C3_9_	Cylinder 1: F1–F3 Cylinder 2: F4–F6 Cylinder 3: F7–F9

**Table 3:** Helium rupture pressures of different samples.

Sample No.	A1	A2	B1	B2	C1	C2
Pressure rise rate (bar/min)	20.8	369.7	10.1	5.1	62.8	30.5
Rupture pressure P_He_ (bar)	569.6	640.8	607.7	616.6	640.3	615.9
Corrected hydrogen rupture pressure P_r_′_He_ (bar)	567.7	643.8	610.3	618.2	641.8	620.0

Corrected helium rupture pressure.

**Table 4:** The P_r_′_He_/P_r_′_H2_ ratio for a three-cylinder body.

Cylinder No: 1.	A3_1_	A3_2_	A3_3_	A3_4_	A3_5_	A3_6_	A3_7_	A3_8_	A3_9_
Pressure rise rate (bar/min)	62.2	30.1	3.7	355.4	239.2	20.0	15.1	6.0	157.4
Corrected helium rupture pressure Pr′_He_ (bar)	531.2	614.6	680.5	839.7	654.4	491.4	562.7	514.8	699.1
Corrected hydrogen rupture pressure Pr′_H2_ (bar)	384.9	399.1	456.7	556.1	448.2	348.5	367.8	352.6	488.9
P_r_′_He_/P_r_′_H2_	1.38	1.54	1.49	1.51	1.46	1.41	1.53	1.46	1.43
The average value	Hydrogen embrittlement index = 1.47								

**Table 5:** The P_r_′_He_/P_r_′_H2_ ratio for the body-shoulder junction.

Cylinder No: 2.	B3_1_	B3_2_	B3_3_	B3_4_	B3_5_	B3_6_	B3_7_	B3_8_	B3_9_
Pressure rise rate (bar/min)	62.2	30.1	3.7	355.4	239.2	20.0	15.1	6.0	157.4
Corrected helium rupture pressure Pr′_He_ (bar)	587.1	659.0	761.4	845.3	680.8	602.3	580.5	490.0	739.2
Corrected hydrogen rupture pressure Pr′_H2_ (bar)	360.2	399.4	440.1	556.1	428.2	356.4	367.4	312.1	453.5
P_r_′_He_/P_r_′_H2_	1.63	1.65	1.73	1.52	1.59	1.69	1.58	1.57	1.63
The average value	Hydrogen embrittlement index = 1.62								

**Table 6:** The P_r_′_He_/P_r_′_H2_ ratio of the shoulder of the three cylinders.

Cylinder No: 3.	C3_1_	C3_2_	C3_3_	C3_4_	C3_5_	C3_6_	C3_7_	C3_8_	C3_9_
Pressure rise rate (bar/min)	62.2	30.1	3.7	355.4	239.2	20.0	15.1	6.0	157.4
Corrected helium rupture pressures Pr′_He_ (bar)	695.2	706.9	850.2	925.1	848.6	588.6	662.0	643.3	911.5
Corrected hydrogen rupture pressure Pr′_H2_ (bar)	377.8	399.4	440.5	486.9	458.7	328.8	359.8	342.2	498.1
P_r_′_He_/P_r_′_H2_	1.84	1.77	1.93	1.90	1.85	1.79	1.84	1.88	1.83
The average value	Hydrogen embrittlement index = 1.85								

**Table 7:** The outcome of the tensile test.

Cylinder Body	D2	D3	D4	D5	D6	D7	D8	D9
Tensile test value	843	827	839	832	829	832	833	828
Yield strength	684	692	687	664	710	707	692	693
Elongation	29	30	26	27	31	30	26	32
Hydrogen embrittlement index	1.54	1.49	1.51	1.46	1.41	1.53	1.46	1.43
**The junction (body and shoulder)**	**F2**	**F3**	**F4**	**F5**	**F6**	**F7**	**F8**	**F9**
Tensile test value	852	851	842	861	858	847	849	851
Yield strength	723	679	712	706	721	724	690	702
Elongation	31	30	32	28	27	33	29	31
Hydrogen embrittlement index	1.65	1.73	1.52	1.59	1.69	1.58	1.57	1.63
**Shoulder**	**E2**	**E3**	**E4**	**E5**	**E6**	**E7**	**E8**	**E9**
Tensile test value	890	908	896	858	867	860	851	867
Yield strength	727	735	708	667	679	701	699	710
Elongation	33	32	28	31	26	34	27	29
Hydrogen embrittlement index	1.77	1.93	1.9	1.85	1.79	1.84	1.88	1.83

## Data Availability

Not applicable.
